# Supplementary material for: An experimentally validated approach to automated biological evidence generation in drug discovery using knowledge graphs
Source: Nat Commun. 2024 Jul 8;15:5703. doi: 10.1038/s41467-024-50024-6 (PMC11231212; doi:10.1038/s41467-024-50024-6)
Supplement: Supplementary file 1 — Supplementary Information [file 41467_2024_50024_MOESM1_ESM.pdf]

Supplementary Tables

**Data source**	**Public**	**Commercial/ Licence**	**Version**
Drugbank		√	version 5.1.10 as of Sep 2023
ChemBL	√		ChEMBL 33
Pharmaprojects		√	1.60.0.0
KEGG		√	version 107.1 as of Aug 2023
Uniprot	√		2023_03
HGNC	√		as of Sep 2023
NCBI	√		version 256 as of Sep 2023
CTD		√	as of Aug 2023
NDF-RT	√		as of Feb 2018
Orphanet	√		as of Jun 2023
Genomics England Panel App	√		as of Sep 2023
OpenTargets	√		23.06
OMIM		√	as of Sep 2023
Reactome	√		as of June 2023
SIDER		√	4.1 as of Oct 2015
Wikipathways	√		as of Aug 2023
MONDO	√		version 2021-08-02
MesH	√		as of Apr 2020
Human Phenotype Ontology	√		version 2023-09-01

Supplementary Table 1. List of public and commercial data sources and their versions used in the Healx KG

Disease – *parent* – Disease
Phenotype – *parent* – Phenotype
Pathway – *involves* – Disease
Compound – *involves* – Pathway
Compound – *involves* – Mechanism
Mechanism – *targets* – Protein
Compound - *in trial for* – Disease
Disease – *presents* – Phenotype
Gene – *associates* – Disease
Compound – *treats* – Disease
Compound – *inhibits* – Gene
Compound – *member* – ATC
Compound – *activates* – Gene
Pathway – *targets* – Pathway
Protein – *codes* – Gene
Compound – *binds* – Gene
Gene – *participates* – Pathway
Gene – *regulates* – Gene
Disease – *ancestor* – Disease
Disease – *descendent* – Disease
Compound – *causes* – Disease

Compound – *in in vivo preclinical trial for* – Disease
Compound – *has orphan designation for* – Disease
Compound – *in in vitro preclinical trial for* – Disease
Compound – *in in vivo preclinical trial effect mitigates* – Disease
Compound – *in in vivo preclinical trial effect no change* – Disease
Compound – *in in vitro preclinical trial effect mitigates* – Disease
Compound – *in in vivo preclinical trial effect exacerbates* – Disease
Compound – *in in vivo preclinical trial effect unknown* – Disease

Supplementary Table 2. List of edge types in Healx KG

Compound <- *involves* – Pathway – *involves* -> Disease
Compound – *treats* -> Phenotype <- *presents* – Disease
Compound – *treats* -> Phenotype <- *presents* – Disease
Compound – *treats* -> Disease <- *ancestor* – Disease
Compound – *inhibits* -> Gene <- *associates* – Disease
Compound – *inhibits* -> Gene <- *regulates* – Gene <- *associates* – Disease
Compound – *inhibits* -> Gene <- *regulates* – Gene <- *regulates* – Gene <- *associates* – Disease
Compound – *inhibits* -> Gene – *inhibits* -> Gene – *in trial for* -> Disease
Compound – *inhibits* -> Gene – *binds* -> Compound – *in trial for* -> Disease
Compound – *in trial for* -> Disease – *ancestor* -> Disease
Compound – *in trial for* -> Disease <- *involves* – Pathway – *involves* -> Disease
Compound – *in trial for* -> Disease – *associates* -> Gene <- *associates* – Disease
Compound – *in trial for* -> Disease <- *in trial for* – Compound – *treats* -> Disease
Compound – *in trial for* -> Disease <- *treats* – Compound – *treats* -> Disease <- *ancestor* – Disease
Compound – *in trial for* -> Disease – *ancestor* -> Disease <- *treats* – Compound – *treats* -> Disease
Compound – *in trial for* -> Disease <- *treats* – Compound – *inhibits* -> Gene <- *associates* – Disease
Compound – *activates* -> Gene <- *participates* – Pathway – *involves* -> Disease
Compound – *activates* -> Gene <- *binds* – Compound – *in trial for* -> Disease
Compound – *activates* -> Gene <- *associates* – Disease
Compound – *activates* -> Gene <- *activates* – Compound – *in trial for* -> Disease
Compound – *activates* -> Gene <- *inhibits* – Compound – *treats* -> Disease
Compound – *treats* -> Disease – *ancestor* -> Disease
Compound – *treats* -> Disease – *associates* -> Gene <- *associates* – Disease
Compound – *treats* -> Disease <- *in trial for* – Compound – *binds* -> Gene <- *associates* – Disease
Compound – *treats* -> Disease <- *treats* – Compound – *treats* -> Disease
Compound – *treats* -> Disease <- *treats* – Compound – *in trial for* -> Disease
Compound – *treats* -> Phenotype <- *causes* – Compound – *treats* -> Disease
Compound – *treats* -> Disease – *ancestor* -> Disease <- *involves* – Pathway – *involves* -> Disease
Compound – *treats* -> Disease – *ancestor* -> Disease <- *in trial for* – Compound – *treats* -> Disease
Compound – *treats* -> Disease – *ancestor* -> Disease <- *treats* – Compound – *treats* -> Disease
Compound – *treats* -> Disease <- *in trial for* – Compound – *treats* →Disease <- *ancestor* – Disease
Compound – *treats* -> Disease <- *treats* – Compound <- *involves* – Pathway – *involves* -> Disease
Compound – *treats* -> Disease <- *involves* – Pathway – *involves* -> Compound – *treats* -> Disease
Compound – *treats* -> Disease – *associates* -> Gene – *participates* -> Pathway – *involves* -> Disease
Compound – *treats* -> Phenotype <- *parent* – Phenotype <- *treats* – Compound – *in trial for* -> Disease
Compound – *treats* -> Phenotype <- *parent* – Phenotype <- *treats* – Compound – *treats* -> Disease
Compound – *treats* -> Phenotype <- *presents* – Disease <- *treats* – Compound – *treats* -> Disease
Compound – *treats* -> Disease <- *in trial for* – Compound – *inhibits* -> Gene <- *associates* – Disease
Compound – *treats* -> Disease – *presents* -> Phenotype <- *presents* – Disease
Compound – *treats* -> Disease – *presents* -> Phenotype <- *parent* – Phenotype <- *presents* – Disease
Compound – *treats* -> Disease <- *involves* – Pathway <- *participates* – Gene – *associates* -> Disease

Compound – *in trial for* -> Disease – *ancestor* -> Disease <- *involves* – Pathway – *involves* -> Disease
Compound – *in trial for* -> Disease <- *involves* – Pathway – *involves* -> Compound – *treats* -> Disease
Compound – *in trial for* -> Disease <- *in trial for* – Compound – *binds* -> Gene <- *associates* – Disease
Compound – *in trial for* -> Disease – *associates* -> Gene – *participates* -> Pathway – *involves* -> Disease
Compound – *in trial for* -> Disease – *associates* -> Gene <- *inhibits* – Compound – *treats* -> Disease
Compound – *binds* -> Gene <- *associates* – Disease
Compound – *binds* -> Gene <- *binds* – Compound – *binds* -> Gene <- *associates* – Disease
Compound – *binds* -> Gene – *participates* -> Pathway <- *participates* – Gene <- *associates* – Disease
Compound – *member* -> ATC <- *member* – Compound – *treats* -> Disease
Compound – *member* -> ATC <- *member* – Compound – *in trial for* -> Disease
Compound <- *has orphan designation for* – Disease <- *in trial for* – Compound – *in trial for* -> Disease
Compound <- *has orphan designation for* – Disease <- *treats* – Compound – *in trial for* -> Disease
Compound <- *involves* – Pathway – *involves* -> Compound – *treats* -> Disease <- *ancestor* – Disease
Compound <- *involves* – Pathway <- *participates* – Gene <- *inhibits* – Compound – *in trial for* -> Disease
Compound <- *involves* – Pathway – *involves* -> Disease <- *involves* – Pathway – *involves* -> Disease
Compound – *involves* -> Mechanism <- *involves* – Compound <- *involves* – Pathway – *involves* -> Disease
Compound – *involves* -> Mechanism <- *involves* – Compound – *in trial for* -> Disease
Compound <- *involves* – Pathway – *involves* -> Compound <- *involves* – Pathway – *involves* -> Disease
Compound <- *involves* – Pathway <- *participates* – Gene – *regulates* -> Gene <- *associates* – Disease
Compound <- *involves* – Pathway <- *participates* – Gene <- *associates* – Disease
Compound <- *involves* – Pathway <- *participates* – Gene <- *inhibits* – Compound – *in trial for* -> Disease
Compound – *involves* -> Mechanism – *targets* -> Protein – *codes* -> Gene <- *associates* – Disease

Supplementary Table 3. Ground truth rules curated for Parkinson's disease and Cystic Fibrosis by drug discovery scientists. Rules curated for both diseases are combined in this table since there are overlaps.

Compound – *in trial for* -> Disease – *associates* -> Gene <- *associates* – Disease
Compound – *inhibits* -> Gene <- *binds* – Compound – *in trial for* -> Disease
Compound – *inhibits* -> Gene <- *inhibits* – Compound – *in trial for* -> Disease
Compound <- *has orphan designation for* – Disease <- *in trial for* – Compound – *in trial for* -> Disease
Compound <- *has orphan designation for* – Disease – *presents* -> Phenotype <- *presents* – Disease
Compound <- *has orphan designation for* – Disease <- *in trial for* – Compound – *treats* -> Phenotype <- *presents* – Disease
Compound <- *involves* – Pathway <- *participates* – Gene <- *associates* – Disease <- *ancestor* – Disease
Compound <- *involves* – Pathway – *involves* -> Compound <- *has orphan designation for* – Disease
Compound – in trial for -> Disease - *has orphan designation for → Compound – in trial for* -> Disease
Compound <- *involves* – Pathway – *involves* -> Disease <-treats - Compound – *in trial for* -> Disease
Compound <- *has orphan designation for* – Disease <- *in trial for* – Compound – *in trial for* -> Disease
Compound <- *has orphan designation for* – Disease <- *ancestor* – Disease <- *in trial for* – Compound – *treats* -> Disease
Compound – *inhibits* -> Gene <- *associates* – Disease <- *in trial for* – Compound – *treats* -> Disease

Compound – *treats* -> Disease <- *ancestor* – Disease <- *in trial for* – Compound – *in trial for* -> Disease
Compound <- *has orphan designation for* – Disease <- *in trial for* – Compound <- *involves* – Pathway – *involves* -> Disease
Compound – in trial for -> Disease <- *ancestor* – Disease <-treats - Compound – *treats* -> Disease
Compound – *involves* -> Mechanism <- *involves* – Compound – *in trial for* -> Disease
Compound <- *has orphan designation for* – Disease <- *in trial for* – Compound <- *has orphan designation for* – Disease
Compound – in trial for -> Disease – presents -> Phenotype <- presents – Disease
Compound <- *involves* – Pathway – *involves* -> Disease <-treats - Compound – *treats* -> Disease
Compound – *in trial for* -> Disease <- *ancestor* – Disease <- *in trial for* – Compound – *treats* -> Disease
Compound – treats -> Phenotype <- treats – Compound – *treats* -> Disease
Compound – treats -> Phenotype – parent -> Phenotype <- treats – Compound – *in trial for* -> Disease
Compound – *in trial for* -> Disease <-treats – Compound – *in trial for* -> Disease
Compound – *in trial for* -> Disease – *associates* -> Gene <- binds – Compound <- *has orphan designation for* – Disease
Compound <- *has orphan designation for* – Disease - *has orphan designation for → Compound – treats* -> Phenotype <- presents – Disease
Compound – *treats* -> Disease – presents -> Phenotype <- *parent* – Phenotype <- presents – Disease
Compound – *inhibits* -> Gene <- *associates* – Disease <- treats – Compound – treats -> Disease
Compound – *involves* -> Mechanism <- *involves* – Compound – *treats* -> Disease – *ancestor* -> Disease
Compound <- *has orphan designation for* – Disease – *ancestor* -> Disease – in trial for - Compound – in trial for → Disease
Compound – *inhibits* -> Gene <- binds – Compound – treats -> Phenotype <-presents – Disease
Compound <- *has orphan designation for* – Disease – *has orphan designation for* → Compound – in trial for -> Disease
Compound <- *has orphan designation for* – Disease – presents -> Phenotype <- treats – Compound – in trial for → Disease
Compound – *binds* -> Gene – participates -> Pathway – participates – Gene – associates -> Disease
Compound – treats -> Phenotype <-treats – Compound – in trial for → Disease
Compound – *treats* -> Disease – *ancestor* -> Disease <- in trial for – Compound –- treats -> Disease
Compound – *in trial for* -> Disease – *has orphan designation for* → Compound – involves -> Pathway – involves -> Disease
Compound – *in trial for* -> Disease – *ancestor* -> Disease <- associates – Gene – associates -> Disease
Compound – inhibits -> Gene – associates -> Disease <- treats – Compound – in trial for → Disease
Compound – *treats* -> Disease <- associates – Gene – regulates -> Gene – associates → Disease
Compound – *treats* -> Disease <- in trial for – Compound – in trial for → Disease
Compound – *treats* -> Disease – presents -> Phenotype <-treats – Compound – in trial for -> Disease
Compound – *in trial for* -> Disease <- in trial for – Compound –- treats -> Disease
Compound – *treats* -> Disease – presents -> Phenotype <- presents – Disease
Compound – *treats* -> Disease <-associates – Gene – participates -> Pathway – involves -> Disease
Compound – *inhibits* -> Gene <- *inhibits* – Compound – *treats* -> Phenotype <-presents – Disease

Supplementary Table 4. Automatically generated rules after filtering for Fragile X Syndrome

Compound	Total possible 2-hop paths	Total filtered paths	Reduction %
Amantadine	41279	72	99.83%
Orphenadrine	23114	23	99.9`5
Pergolide	55966	152	99.73%
Bromodiphenhydramine	4820	2	99.96%
Ropinirole	36492	123	99.66%
Droxidopa	29492	22	99.93%
Ioflupane	5812	9	99.85%
Pimavanserin	21798	37	99.83%
Methixene	15134	6	99.96%
Pramipexole	34036	73	99.79%
Procyclidine	10438	5	99.95%
Opicapone	3897	36	99.08%
Trihexyphenidyl	12304	8	99.93%
Melevodopa	787	1	99.87%
Tolcapone	13118	45	99.66%
Entacapone	11438	2596	77.3%
Ethopropazine	8977	4	99.96%
Budipine	7014	10	99.86%
Istradefylline	8280	21	99.75%
Apomorphine	52492	140	99.73%
Cabergoline	58621	27143	53.7%
Piribedil	9077	36	99.6%
Selegiline	22065	85	99.61%
Dopamine	60926	123	99.8%
Levodopa	46234	171	99.63%
Azilect	5800	15	99.74%
Bromocriptine	70702	186	99.74%
Benserazide	3758	12	99.68%
Rasagiline	18246	33	99.82%
Carbidopa	8903	22	99.75%
Biperiden	16838	14	99.92%
Safinamide	12425	46	99.63%
Rivastigmine	27097	2457	90.93%
Rotigotine	45879	122	99.73%

Supplementary Table 5. Reduction in evidence chains space with AnyBURL and the auto-filtering pipeline with subgraph of Healx KG shown for 34 predicted approved treatments in Parkinson's disease. Source data are provided as a Source Data file.

Supplementary Figures

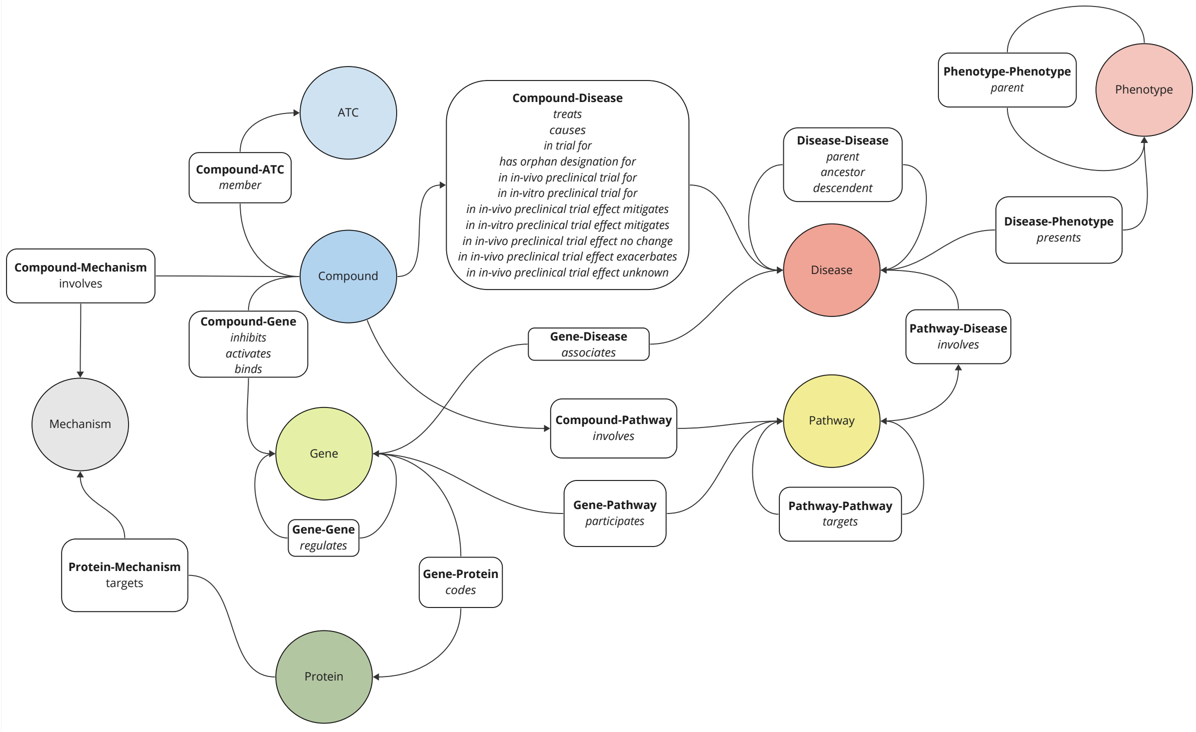

Supplementary Figure 1. **Meta graph of Healx KG.** The meta graph shows the 8 different node types and 29 edge types and how they are connected in the Healx KG

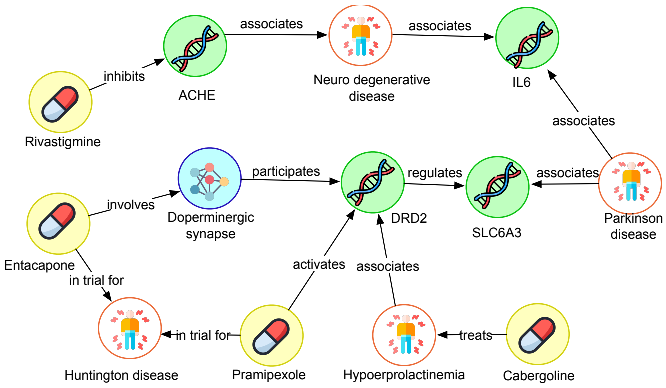

Supplementary Figure 2. **Evidence chains for Parkinson's Disease with Healx KG sub-graph.** Few interesting evidence chains observed for Parkinson's disease with the auto-filtering pipeline in the subgraph of Healx KG for Rivastigmine, Entacapone and Cabergoline. Yellow symbol indicates the type compound, white indicates disease, green indicates gene and blue indicates pathway. Source data are provided as a Source Data file.
