# Supplementary material for: An experimentally validated approach to automated biological evidence generation in drug discovery using knowledge graphs
Source: Nat Commun. 2024 Jul 8;15:5703. doi: 10.1038/s41467-024-50024-6 (PMC11231212; doi:10.1038/s41467-024-50024-6)
Supplement: Supplementary file 2 — Reporting Summary [file 41467_2024_50024_MOESM2_ESM.pdf]

nature portfolio

Corresponding author(s): Saatviga Sudhahar

Last updated by author(s): 24/06/2024

Reporting Summary

Nature Portfolio wishes to improve the reproducibility of the work that we publish. This form provides structure for consistency and transparency in reporting. For further information on Nature Portfolio policies, see our Editorial Policies and the Editorial Policy Checklist.

Statistics

For all statistical analyses, confirm that the following items are present in the figure legend, table legend, main text, or Methods section.

n/a	Confirmed	
x		The exact sample size (*n*) for each experimental group/condition, given as a discrete number and unit of measurement
x		A statement on whether measurements were taken from distinct samples or whether the same sample was measured repeatedly
x		The statistical test(s) used AND whether they are one- or two-sided<br>*Only common tests should be described solely by name; describe more complex techniques in the Methods section.*
x		A description of all covariates tested
x		A description of any assumptions or corrections, such as tests of normality and adjustment for multiple comparisons
	x	A full description of the statistical parameters including central tendency (e.g. means) or other basic estimates (e.g. regression coefficient) AND variation (e.g. standard deviation) or associated estimates of uncertainty (e.g. confidence intervals)
	x	For null hypothesis testing, the test statistic (e.g. *F*, *t*, *r*) with confidence intervals, effect sizes, degrees of freedom and *P* value noted<br>*Give P values as exact values whenever suitable.*
x		For Bayesian analysis, information on the choice of priors and Markov chain Monte Carlo settings
x		For hierarchical and complex designs, identification of the appropriate level for tests and full reporting of outcomes
x		Estimates of effect sizes (e.g. Cohen's *d*, Pearson's *r*), indicating how they were calculated

Our web collection on statistics for biologists contains articles on many of the points above.

Software and code

Policy information about availability of computer code

Data collection	No software was used for data collection
Data analysis	No software used

For manuscripts utilizing custom algorithms or software that are central to the research but not yet described in published literature, software must be made available to editors and reviewers. We strongly encourage code deposition in a community repository (e.g. GitHub). See the Nature Portfolio guidelines for submitting code & software for further information.

Data

Policy information about availability of data

All manuscripts must include a data availability statement. This statement should provide the following information, where applicable:

- Accession codes, unique identifiers, or web links for publicly available datasets
- A description of any restrictions on data availability
- For clinical datasets or third party data, please ensure that the statement adheres to our policy

In Supplementary Table 1 we provide details of both public and commercial data sources used in Healx KG. CTD100, SIDER108, DrugBank101, KEGG104, OMIM112 and Pharmaprojects are commercial data sources. CTD100 data can be used only for research and educational purposes, and any Commercial users are required to purchase a license to access data from the CTD website. SIDER108 data is licenced under a creative commons Attribution-Noncommercial-Share Alike 4.0 License. For commercial use or customized versions, license should be obtained from biobyte solutions GmbH. Use and re-distribution of the content of DrugBank101 for any purpose requires a license. Academic users may apply for a free license for certain use cases and all other users require a paid license. KEGG104 database is

available for academic use but any commercial use requires a license. Use of OMIM112 is provided free of charge to any individual for personal use, for educational or scholarly use, or for research purposes through the front end of the database. Commercial users who want to download all or part of OMIM must obtain a license by paying applicable licensing fees to and entering into a license agreement with Johns Hopkins University (JHU). Pharmaprojects comes with a commercial license granting full access to their APIs. We have shared a subgraph of the Healx KG data created for reproducibility purposes and is available in github, https://github.com/healx/automated-biological-evidence-generation-in-drug-discovery128. We have shared the experimental results from this subgraph showing a few interesting evidence chains generated for Parkinson's disease in Supplementary Fig. 2 and the percentage of reduction achieved in Supplementary Table 5. The source data for this is provided as a Source Data file. The raw sequence (RNA-seq) data used in the Fragile X study has been deposited in the NCBI Sequence Read Archive (SRA)) under BioProject PRJNA1096445 titled 'Brain-specific gene expression changes in FXS mouse model after Sulindac or Ibudilast treatment'.

The list of curated rules from the complete set given by AnyBURL as given by drug discovery scientists for both Parkinson's disease and Cystic Fibrosis are included in Supplementary Table 3. The rules set produced for predictions in both diseases have overlaps, hence they are presented in a single table. We also present FXS rules separately in Supplementary Table 4 which were automatically generated by the pipeline before evidence chain generation. The curated list was only used in Parkinson's and Cystic fibrosis to check if the automatic filtering retained these useful rules. All rules shared in the supplementary files are from the full Healx KG.

Research involving human participants, their data, or biological material

Policy information about studies with human participants or human data. See also policy information about sex, gender (identity/presentation), and sexual orientation and race, ethnicity and racism.

Reporting on sex and gender	N/A
Reporting on race, ethnicity, or other socially relevant groupings	N/A
Population characteristics	N/A
Recruitment	N/A
Ethics oversight	N/A

Note that full information on the approval of the study protocol must also be provided in the manuscript.

Field-specific reporting

Please select the one below that is the best fit for your research. If you are not sure, read the appropriate sections before making your selection.

☒ Life sciences ☐ Behavioural & social sciences ☐ Ecological, evolutionary & environmental sciences

For a reference copy of the document with all sections, see nature.com/documents/nr-reporting-summary-flat.pdf

Life sciences study design

All studies must disclose on these points even when the disclosure is negative.

Sample size	The Healx KG subgraph we have shared for reproducibility purpose has 129,501 edges and 37,331 nodes. The sampling was done to create a minimal viable graph capable of producing results for Parkinson's disease. From the full Healx KG, we sampled data that was one hop away from the node Parkinson's disease and the 45 approved treatment compounds. Treatment edges between these compounds and the node Parkinson's disease was removed.
Data exclusions	No specific exclusions
Replication	We share a sub-graph of the Healx KG without any proprietary data and present experimental results from this graph in Supplementary Table 5 and Supplementary Figure 2 which can be reproduced. We confirm that the source code shared with the manuscript was used to generate the results from the study including reproducible results in the Supplementary information.
Randomization	This is not relevant since we considered sampling one hop away from the Parkinson's disease and the 45 compound nodes.
Blinding	This is not relevant

Reporting for specific materials, systems and methods

We require information from authors about some types of materials, experimental systems and methods used in many studies. Here, indicate whether each material, system or method listed is relevant to your study. If you are not sure if a list item applies to your research, read the appropriate section before selecting a response.

Materials & experimental systems

n/a	Involved in the study
☒	☐ Antibodies
☒	☐ Eukaryotic cell lines
☒	☐ Palaeontology and archaeology
☐	☒ Animals and other organisms
☒	☐ Clinical data
☒	☐ Dual use research of concern
☒	☐ Plants

Methods

n/a	Involved in the study
☒	☐ ChIP-seq
☒	☐ Flow cytometry
☒	☐ MRI-based neuroimaging

Animals and other research organisms

Policy information about studies involving animals; ARRIVE guidelines recommended for reporting animal research, and Sex and Gender in Research

Laboratory animals	Fmr1 Knockout(KO), strain 004624, 10 male mice aged 2 months were used for each treatment group
Wild animals	The study did not involve wild animals.
Reporting on sex	In the Fragile X study due to the disease being an X-linked inherited disorder, with the most severe phenotype persisting in males, we only tested in male mice.
Field-collected samples	The study did not involve field-collected samples
Ethics oversight	The research was carried out by a third party CRO in line with the requirements of the United Kingdom Animals (Scientific Procedures) Act, 1986.

Note that full information on the approval of the study protocol must also be provided in the manuscript.

Plants

Seed stocks	N/A
Novel plant genotypes	N/A
Authentication	N/A
